# SECOM: A Novel Hash Seed and Community Detection Based-Approach for Genome-Scale Protein Domain Identification

**DOI:** 10.1371/journal.pone.0039475

**Published:** 2012-06-28

**Authors:** Ming Fan, Ka-Chun Wong, Taewoo Ryu, Timothy Ravasi, Xin Gao

**Affiliations:** 1 Mathematical and Computer Sciences and Engineering Division, King Abdullah University of Science and Technology (KAUST), Thuwal, Saudi Arabia; 2 Department of Computer Science, University of Toronto, Toronto, Ontario, Canada; 3 Terrence Donnelly Centre for Cellular and Biomolecular Research, University of Toronto, Toronto, Ontario, Canada; 4 Division of Chemical and Life Sciences and Engineering, Computational Bioscience Research Center, King Abdullah University of Science and Technology (KAUST), Thuwal, Saudi Arabia; National Institute for Medical Research, Medical Research Council, United Kingdom

## Abstract

With rapid advances in the development of DNA sequencing technologies, a plethora of high-throughput genome and proteome data from a diverse spectrum of organisms have been generated. The functional annotation and evolutionary history of proteins are usually inferred from domains predicted from the genome sequences. Traditional database-based domain prediction methods cannot identify novel domains, however, and alignment-based methods, which look for recurring segments in the proteome, are computationally demanding. Here, we propose a novel genome-wide domain prediction method, SECOM. Instead of conducting all-against-all sequence alignment, SECOM first indexes all the proteins in the genome by using a hash seed function. Local similarity can thus be detected and encoded into a graph structure, in which each node represents a protein sequence and each edge weight represents the shared hash seeds between the two nodes. SECOM then formulates the domain prediction problem as an overlapping community-finding problem in this graph. A backward graph percolation algorithm that efficiently identifies the domains is proposed. We tested SECOM on five recently sequenced genomes of aquatic animals. Our tests demonstrated that SECOM was able to identify most of the known domains identified by InterProScan. When compared with the alignment-based method, SECOM showed higher sensitivity in detecting putative novel domains, while it was also three orders of magnitude faster. For example, SECOM was able to predict a novel sponge-specific domain in nucleoside-triphosphatase (NTPases). Furthermore, SECOM discovered two novel domains, likely of bacterial origin, that are taxonomically restricted to sea anemone and hydra. SECOM is an open-source program and available at http://sfb.kaust.edu.sa/Pages/Software.aspx.

## Introduction

In recent years, genome sequencing projects have generated a vast amount of biological sequence data. To make use of these data, comparative analysis has often been used to induce meaningful hypotheses through discovery of conserved sequences with regulatory functions and novel genes [1].

Each protein contains domains that have unique functions and can evolve independently of the rest of the protein chain [2]. A domain is generally considered as a compact and semi-independent unit that can fold into a stable, three-dimensional (3D) structure [3]. Molecular evolution gives rise to families of related proteins with similar sequences and structures. Such evolutionary relationships between closely related species can be revealed by comparative analysis of their domains [4], [5].

The prediction of protein domains has long been considered one of the most fundamental steps in deciphering the evolution and functions of proteins as well as species. Domain detection is often closely related to the determination of discrete structural folding units. Various domain prediction methods have been reported in the literature. The existing methods can be classified into two main categories [6], namely template-based methods and *de novo* (or *ab-initio*) methods. The template-based methods identify the similarities between a target sequence and the template sequences in a protein structure database such as Protein Data Bank (PDB) [7]. However, the accuracy of the template-based methods is highly dependent on the quality of the template structures. Therefore, such methods should not be assumed to work well for proteins containing novel domains, especially when they are from less characterized species. On the other hand, the *ab-initio* methods can predict protein domains by taking advantage of various sequence-based features, including sequence profiles, secondary structure predictions, and correlated mutations. Those methods use computational tools, such as neural networks [8], support vector machines [9], [10], and hidden Markov models [11]. However, the accuracy of *ab-initio* domain prediction methods on multidomain proteins is still very low [12].

All these methods have either a well-defined structural database or structure-related features as their foundations. However, structural information is available for only a very tiny fraction of the entire set of proteins. Therefore, there is an increasing need to predict novel domain-specific signatures from protein sequences. Moreover, when the proteome data are given as the input (e.g., proteins from a single genome), more information can be found. Homologous analysis of the sequences is assumed to provide evolutionary, functional, and structural information. The main difference between proteome-scale and single-protein-level domain detection is that a domain is assumed to be a recurring segment of amino acids within the proteome.

Various homologous search approaches have been proposed to solve this problem. The DIVCLUS program [13] performs all-against-all Smith-Waterman pairwise comparisons. The resulting pairs are then merged using single linkage clustering. This method is quite sensitive but computationally expensive. The Domainer algorithm [14] works in a similar manner. It first conducts an all-against-all BLAST search to identify segment pairs with high degrees of homology. These segment pairs are then iteratively merged into consistent clusters. There are two main bottlenecks in the existing all-against-all alignment-based methods. First, after the pairwise alignment, irrelevant domains are clustered into the same domain by the clustering algorithms. For instance, a protein may comprise several different domains or even multiple copies of the same domain. The widely used single linkage-clustering algorithm merges these different domains into one due to the chain effect. Second, the asymptotic runtime of the most efficient method is still 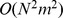, where *N* is the number of proteins in the inquiry dataset and *m* is the maximum length of the proteins in the dataset. This is too slow for the proteome-scale domain detection problem.

To overcome these two bottlenecks, we propose a novel genome-scale domain detection method: SECOM, a hash SEed and COMmunity searching-based domain detection method. Given all the protein sequences from a genome, SECOM efficiently identifies all the sequentially homologous regions that recur within these proteins. SECOM does not conduct all-against-all sequence comparisons. Instead, we assume that the domains of the input protein set have highly conserved segments. The highly conserved segments are not necessarily those sharing identical amino acids, however. They may be those with sequential similarities. SECOM identifies the highly conserved segments by using hash seeds as proposed in a recent study by Li et al. [15]. We then formulate the domain detection problem into a graph representation, in which each node is an input protein sequence and each edge represents the number of hash seeds shared between the two nodes. The problem is to identify all the strongly connected subgraphs. Such subgraphs, however, can overlap because a protein sequence can contain different domains. Therefore, we introduce a clique percolation algorithm to identify the strongly connected subgraphs, i.e., communities, in the graph. Each community corresponds to a domain detected by SECOM. In this way, SECOM is able to identify the overlapping domains. The runtime is nearly-linear to the size of the inputs and quadratic to the number of domains, which is a much smaller number than the size of the input.

## Materials and Methods

### Outline of SECOM

**Figure 1:**
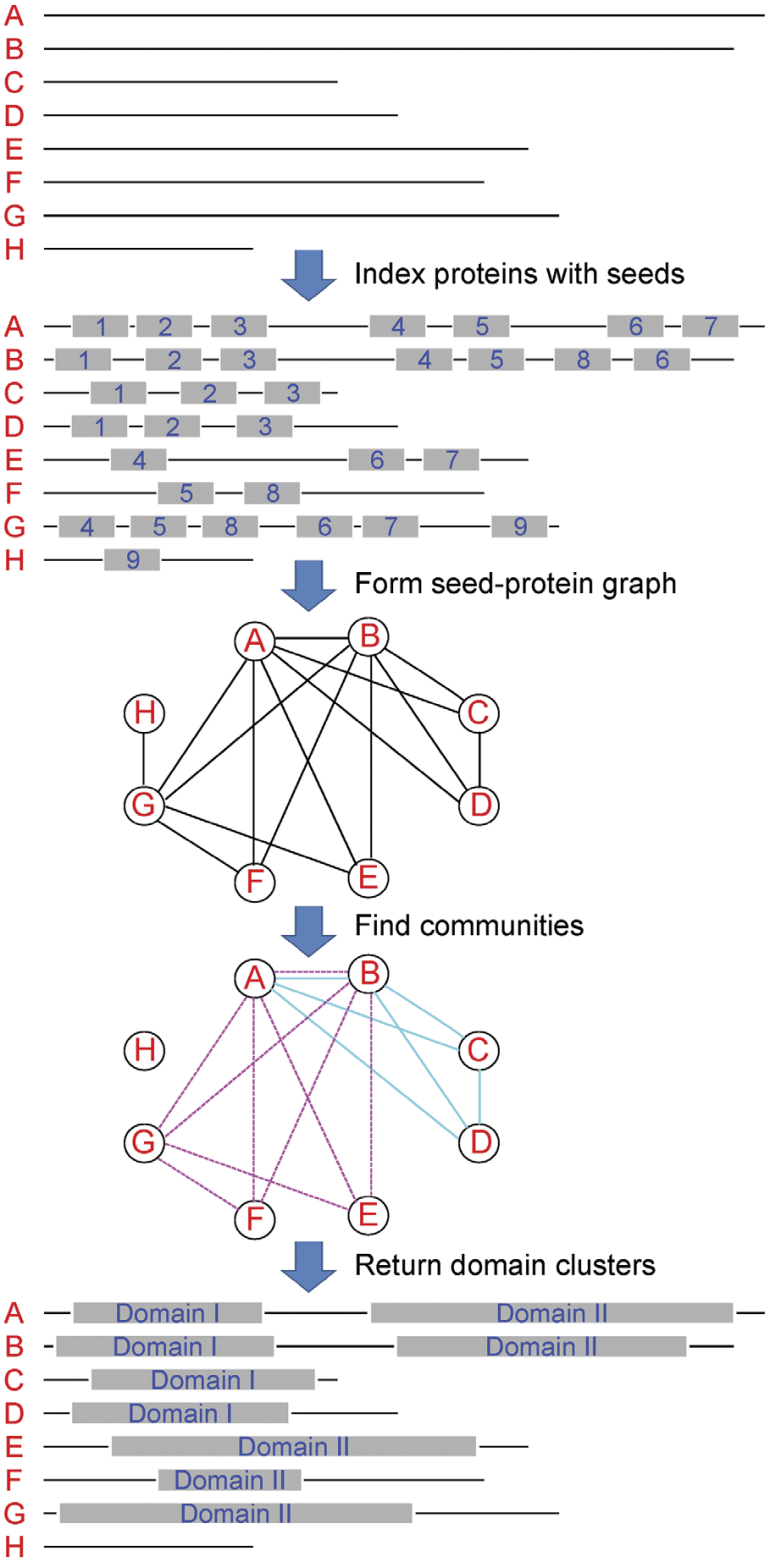
Outline of SECOM. Given a set of protein sequences (“A” to “H”), SECOM first finds all the hash seeds (“1” to “9”) that appear at least twice in this set. A seed-protein graph is then built, in which each node is a protein sequence and two nodes are connected if they share at least one hash seed. The highly connected subgraphs (i.e., communities) are found in this graph. The communities can be overlapping and each of them (“Domain I” and “Domain II”) is a predicted domain cluster by SECOM.

At the foundation of our method is the assumption that if a cluster of protein segments corresponds to the same domain, most pairs of these segments should have at least one small fragment that shares high sequential similarity; i.e., the pairs of segments share hash seeds. The cluster of segments that correspond to a domain is called a domain cluster. The outline of SECOM is illustrated in Figure 1. Given a set of protein sequences, SECOM first identifies the highly conserved fragments, i.e., the hash seeds, which occur at least twice in this set.

After this step, we have a many-to-many mapping between the protein sequences and the hash seeds; i.e., each protein sequence contains some hash seeds and each hash seed corresponds to a number of protein sequences. This mapping can be represented by a graph, where the nodes represent the protein sequences. Two nodes are connected if the two protein sequences share at least one hash seed. The weights of the edges are the numbers of shared seeds. Ideally, a domain is represented by a clique in this graph.

However, due to mutation during evolution, same domains even in the paralogs may share no hash seed. Because of this, we aim at finding strongly connected subgraphs, instead of the cliques. Meanwhile, a protein is usually composed of different domains, which imposes the requirement that the subgraphs can have overlapping nodes and edges in our graph problem formulation. This is equivalent to the problem of finding overlapping communities in complex networks. We propose a backward clique percolation algorithm that efficiently identifies domains in the graph. In the remainder of this section, we introduce the technical details of SECOM.

### Indexing Protein Sequences with Hash Seeds

Li et al. proposed the idea of hash seeds [15]. A hash seed is a short fragment of amino acids. The size of the amino acid alphabets can be either 20 or smaller, such as the classifications proposed in [15]. A hash function is used to calculate the unique hash value for a hash seed, which enables efficient seed matching. Li et al. [15] also demonstrated that hash seed-based homology searches are significantly more sensitive and efficient than exact seed- and spaced seed-based searches. We therefore utilize the hash seed idea to find highly conserved fragments in the input protein sequences.

All the protein sequences are parsed into sliding fragments of length *n* and step size one. The hash function with a large prime base is used to calculate the hash value of each fragment. The amino acid classifications proposed in [15] are used in SECOM as an option for users. A protein sequence is represented by a set of successive 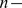
mers and hence as hash seeds. Protein homology searches can be efficiently performed through these hash seeds instead of through amino acids. Two hash seeds generate a hit if and only if they have the same hash value. All of the proteins in the database can thus be indexed in this way. The hash seeds are then stored in a balanced binary search tree according to their hash values, which can be done in 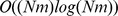 time, where *N* is the number of proteins in the inquiry dataset and *m* is the maximum length of the proteins in the dataset. Further implementation details about the hash seeds can be found in the Materials S1.

### Domain Detection through Clique Percolation

The length of the hash seeds is short compared with the length of the domains. Thus, the protein segments that correspond to a domain are supposed to contain similar sets of hash seeds. The next step is to identify all such groups of segments. We first convert the mapping between the protein sequences and the hash seeds into an undirected graph, in which each node represents a protein sequence and each edge represents the number of shared hash seeds between the two sequences. If there are no common hash seeds between two protein sequences, there is no edge between the two nodes in the graph. Ideally, if a domain does not have mutations in the corresponding proteins, there should be a complete subgraph, i.e., a clique, with the same, high edge weights in the graph connecting those proteins together. However, due to changes during the evolution, the same domain sequence in different proteins may contain different numbers of hash seeds, or even no hash seeds. Therefore, the problem is formulated as finding all the strongly connected subgraphs in the graph. Moreover, since proteins usually contain more than one domain, a practical algorithm must be able to find the overlapping subgraphs.

In graph theory, a subgraph that is more highly connected than other parts of the graph is also called a community. The community-finding problem has received much attention since the seminal paper by Newman [16]. Unfortunately, the overlapping community-finding problem has not been tackled in most of the traditional graph-based or clustering algorithms. In 2005, Palla et al. proposed a clique percolation method for uncovering overlapping communities [17]. They defined the *k*-clique community as a set of nodes belonging to adjacent *k*-cliques, i.e., cliques with *k* nodes. Later, Kumpula et al. proposed a more efficient clique percolation algorithm to find the overlapping *k*-communities [18], for a fixed *k*. Their algorithm works in a sequential manner. This algorithm can detect the overlapping *k*-clique communities in linear time in terms of the number of *k*-cliques in the graph.

However, none of these algorithms can be directly applied to the domain finding problem. Both algorithms require the enumeration of all cliques with sizes smaller than 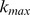, where 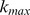 is the size of the largest clique in the graph. This is not practical for proteome-scale domain detection, in which we have a dense graph of about 20,000–70,000 nodes. A populated domain can appear hundreds or even thousands of times in a genome. On the other hand, one may suggest using a small value instead of 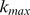 to overcome this issue. However, this will cause irrelevant domains to be merged together due to the chain effects.

Here, we propose a heuristic algorithm that does not enumerate all the small cliques by using the properties of the domain detection problem. First, from our protein sequence-indexing step, we extract and store all the sequences that share the same hash seeds. According to the way our graph is defined, all such sequences are connected to each other and thus form a clique. Second, the more frequently a hash seed appears, the higher the confidence assigned to this seed. Larger cliques therefore have higher confidence.

According to these properties, in order to avoid the chain effect caused by the 2-clique, our algorithm is designed to work in a backward manner. It first eliminates all the edges with weights smaller than a pre-defined threshold. We use two as the default value, which means that two sequences are considered to be homologous if they contain at least two common hash seeds. The algorithm then begins with the largest clique in the graph, i.e., the one that corresponds to the most frequent hash seed. If there are other cliques with the same size 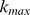 in the graph, our algorithm projects the cliques into 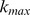 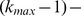
cliques using the same method described in [18]. Each connected component in this projection corresponds to a 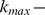
clique community. The communities are then compared with the communities with larger size. If the majority (SECOM uses 70% as the default) of the nodes of the smaller community are shared between the two, these two communities are merged. This procedure continues until no additional merges can be conducted. Our algorithm then checks the clique size in descending order, until size two. For a clique size *k*, if there is no clique with the same size, it can still be merged into a community if at least 70% of the nodes are members of the community.

The overlapping communities can thus be generated through this backward clique percolation algorithm. For the percolation steps in our algorithm, the runtime is linear in terms of the number of cliques, as shown in [18]. For the community merging steps, the worse-case runtime is quadratic in terms of the number of communities, which is usually a much smaller number than the number of nodes or cliques.

Note that SECOM predicts the conserved regions of the domains instead of estimating the exact boundaries of the domains. To predict the boundaries, one can apply the widely used method in local alignment algorithms, which extends the aligned conserved regions in both directions until the alignment score is lower than a certain threshold. Biological features can also be extracted to enhance the prediction accuracy for boundaries. Since these are not the main focus of the paper, we leave it as a user option.

## Results

### Validation of the Proposed Method

To assess the ability of SECOM to identify domains, we ran SECOM on five recently sequenced non-model organism genomes including a sponge [19], hydra [20], sea anemone [21], sea urchin [22], and coral [23], which contain 30,327, 17,398, 27,273, 42,420, and 69,160 annotated protein sequences, respectively. The details about the five proteomes can be found in the Materials S1.

SECOM has three parameters, which are available for the users to set, i.e., the length of the hash seeds (*n*), the threshold for merging two communities (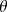), and the amino acid classification. By default, *n* is set to 9, 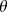 is set to 70%, and the 20 amino acids are classified into 15 groups as described in [15]. The discussion about how the performance varies for different parameter settings can be found in Figures S1, S2, S3, S4, S5, S6, S7, S8, S9, S10 in the Materials S1.

**Figure 2:**
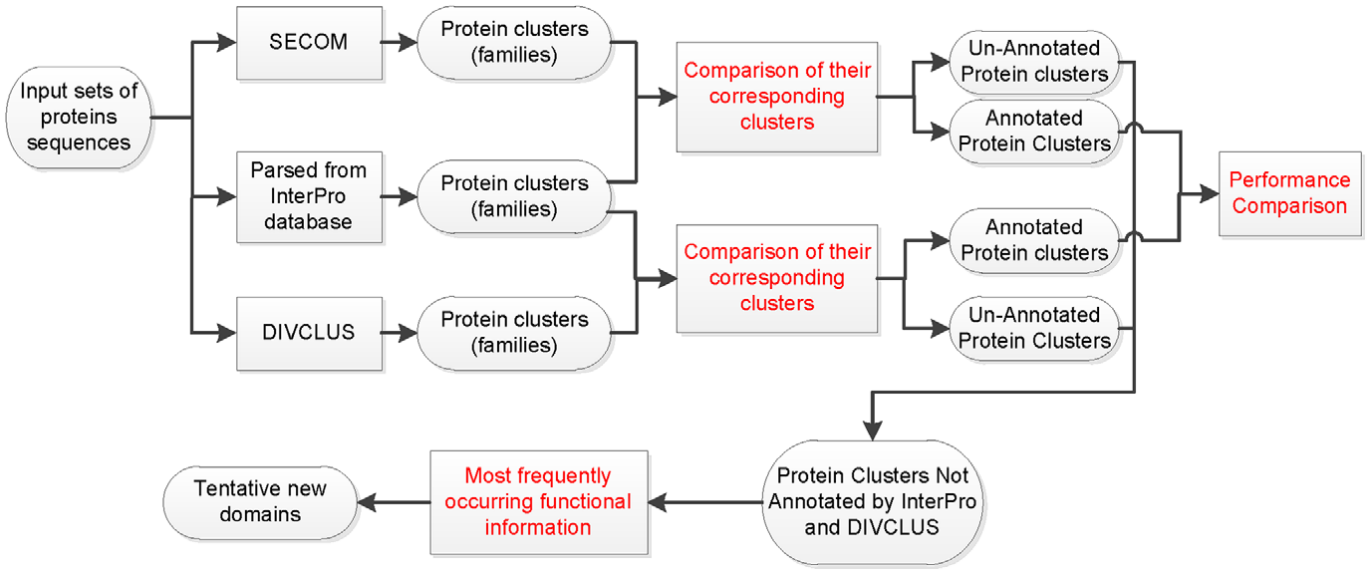
Illustration of the multi-step validation procedure to evaluate the performance of SECOM. SECOM and DIVCLUS are first tested by recovering the Pfam and Superfamily domains annotated by InterProScan. The putative novel domains predicted by SECOM and DIVCLUS are then compared against each other. The putative novel domains predicted only by SECOM are finally analyzed.

To evaluate the performance of SECOM, we conducted a step-by-step validation process by comparing SECOM with both the database-based (i.e., InterProScan) and the alignment-based (i.e., DIVCLUS) domain detection methods. The domains identified by SECOM are first compared with the Pfam and Superfamily domains predicted by InterProScan [24] to evaluate the ability of SECOM to recover the results of the database-based methods. The domains predicted by SECOM but not by InterProScan are deemed as putative novel domains. We then compared the putative novel domains identified by SECOM and DIVCLUS to evaluate the ability of SECOM to recover the results of the alignment-based methods. We further analyzed the putative novel domains that are predicted by SECOM, but not by InterProScan or DIVCLUS. The outline of the validation procedure is illustrated in Figure 2.

### Performance on Recovering Database Annotations

In validating SECOM, we assumed that domain annotations by InterProScan were the “gold standard” and evaluated the ability of SECOM to reproduce the results of InterProScan. We evaluated both cluster-level and in-cluster-level performance. The cluster-level performance measures how many domain clusters are recovered, whereas the in-cluster-level performance measures how many segments in a cluster are recovered. The overall performance metrics for SECOM and DIVCLUS are presented in Table 1, and details of the evaluation criteria can be found in the Materials S1.

**Table 1:** Overall performance of SECOM and DIVCLUS on the five aquatic proteomes.

Species	Sponge		Coral		Hydra		Urchin		Anemone		*Average*	
Method	DIV	SEC	DIV	SEC	DIV	SEC	DIV	SEC	DIV	SEC	DIV	SEC
*recall_clu_*	51.6	57.0	9.4	13.8	57.5	63.0	89.9	97.4	2.2	51.4	42.1	56.5
*precision_clu_*	68.6	62.1	89.6	51.3	70.9	64.1	80.6	77.9	95.6	69.1	81.1	64.9
*F*1*_clu_*	58.9	59.4	17.0	21.7	63.5	63.5	85.0	86.6	4.3	59.0	55.4	60.4
*recall_inClu_*	17.3	17.6	20.7	26.8	22.7	24.7	17.6	17.5	18.7	17.5	19.4	20.8
*precision_inClu_*	98.7	97.0	99.9	99.3	99.2	98.6	99.7	99.3	99.9	99.2	99.5	98.7
*F*1*_inClu_*	29.4	29.8	34.3	42.2	36.9	39.5	29.9	29.8	31.5	29.8	32.5	34.4
Runtime (min)	3660	1.4	1803	0.4	4024	0.7	9371	6.7	2103	1.1	4192.2	2.1

All the recall, precision and F1 score values are percentiles. DIV denotes DIVCLUS and SEC denotes SECOM.

As we can see from Table 1, SECOM has higher recall than DIVCLUS has on both the cluster level and the in-cluster level, whereas DIVCLUS demonstrates higher precision. This is because DIVCLUS uses all-against-all alignment while SECOM is a local seed-based method. It has been demonstrated that seed-based methods are more sensitive than alignment-based methods because they are centered on local homologous regions [25], [26]. The higher precision but lower recall suggests that DIVCLUS tends to find domain segments with high sequential similarities, which usually results in small domain clusters. SECOM, on the other hand, finds more domain clusters and more segments in those clusters, which results in the lower precision. It should be noted that such conclusions are based on the assumption that the InterProScan annotations are ideal. Overall, we showed that the tradeoff between recall and precision for SECOM is better than that for DIVCLUS at both cluster and in-cluster levels, and SECOM is on average 2,000 times faster than DIVCLUS.

We then compared SECOM with DIVCLUS on more details. As test dataset we used the sponge protein repertoire. The comparison results on the other proteomes were similar (data not shown). The sponge proteome contained 30,327 predicted protein sequences. After excluding protein sequences shorter than 20 amino acids, 30,124 sequences were used as input. In total, InterProScan identified 4,091 domain clusters, 2,627 of which contain at least two segments from the sponge protein sequences. Since both SECOM and DIVCLUS required a domain to appear at least twice in the proteome, we considered these 2,627 domains as the “gold standard”.

**Figure 3:**
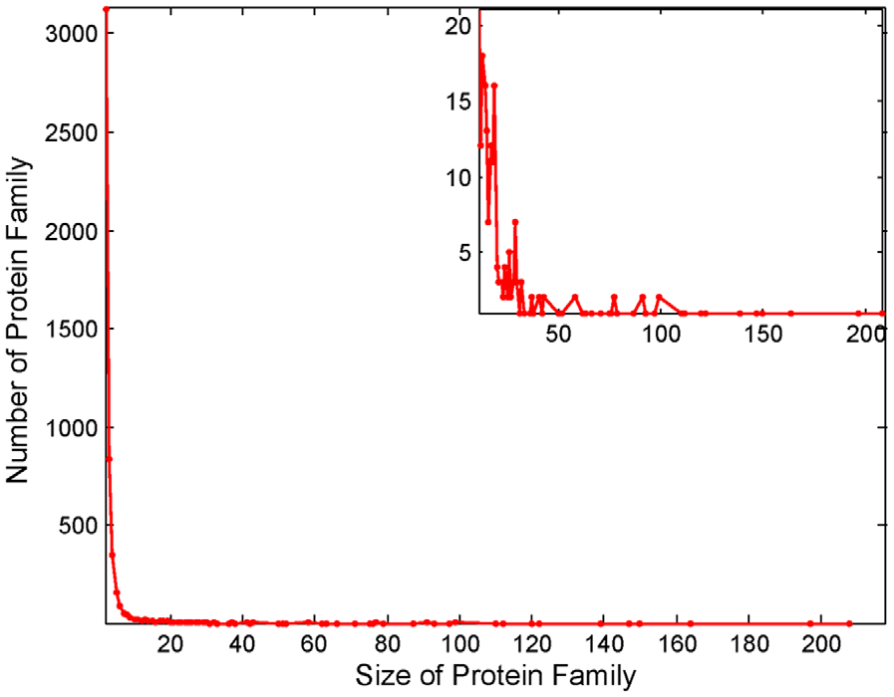
Distribution of cluster sizes for the domain segments predicted by SECOM. The distribution of the clusters with larger sizes, containing at least 11 segments, are enlarged as the inset.

SECOM predicted 4,919 domains for the sponge proteome, whereas DIVCLUS predicted 3,840 domains. Most of the SECOM predicted domains (90.0%) contained less than six segments with the largest domain containing 207 segments. The distribution of size of the domain clusters is shown in Figure 3. As expected, cluster size follows a power law distribution. In total, 62% (3,055/4,919) domains predicted by SECOM and 68% (2,634/3,840) domains predicted by DIVCLUS matched InterProScan’s results. The average size of the clusters of the SECOM-recovered domains was four, whereas the average size for the corresponding clusters was 51 when predicted by InterProScan. This suggests that the domains annotated by InterProScan tend to have larger cluster size. As discussed earlier, SECOM predicts more InterProScan domains than DIVCLUS does (Table 1). The tradeoff between the recall and the precision for SECOM is better than that for DIVCLUS as demonstrated by the higher F1 score. SECOM is also three orders of magnitude faster than DIVCLUS.

We further tried to evaluate the revised performance of SECOM and DIVCLUS by considering the annotations of InterProScan as unperfect. First, we removed the segments that did not share at least two hash seeds with other segments in the same cluster from the clusters of segments annotated by InterProScan. Then, when a predicted domain cluster was compared with an annotated domain cluster, the segments that shared at least two hash seeds with all the other segments in the predicted cluster were considered true positive segments. Following this, we compared the revised performance of SECOM and DIVCLUS, as shown in Table 2 (and Table S1). As we can see, all the revised recall and precision values for both methods are higher than the values obtained by using InterProScan output as the “gold standard”, with SECOM predicting 69.2% of the InterProScan domains. In these clusters, almost all the segments (99.8%) detected by SECOM share high sequential similarities. On average, 76.7% of the segments annotated by InterProScan are detected by SECOM and grouped into the correct clusters. The additional domain clusters predicted by SECOM but not by InterProScan are considered as putative novel domains.

**Table 2:** Revised performance SECOM and DIVCLUS on the sponge proteome.

	DIV	SEC
*recall_clu_*	61.6	69.2
*precision_clu_*	70.3	63.8
*F*1*_clu_*	65.8	66.4
*recall_inClu_*	76.2	76.7
*precision_inClu_*	99.7	99.8
*F*1*_inClu_*	86.4	86.7
Runtime (min)	3660	1.4

All the recall, precision and F1 score values are given as percentiles. DIV denotes DIVCLUS and SEC denotes SECOM.

**Figure 4:**
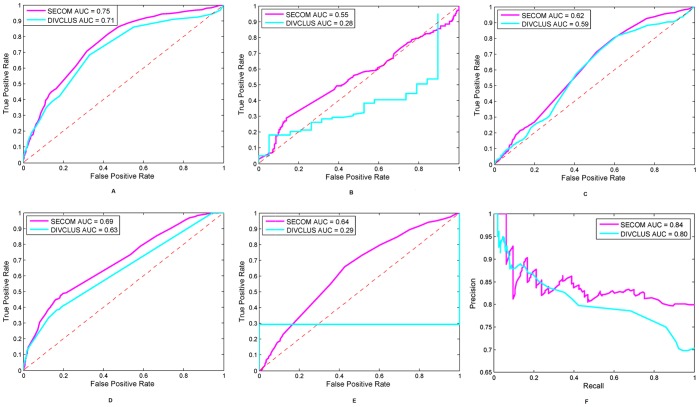
Performance with varying thresholds. (A)–(E). ROC curves for SECOM (magenta) and DIVCLUS (cyan) on sponge, coral, hydra, urchin and sea anemone, respectively. (F). Precision-recall curves for SECOM (magenta) and DIVCLUS (cyan) on sponge. The ROC plots suggest that SECOM provides a better overall performance. At small false positive rate, SECOM has very similar but slightly lower AUC, i.e., AUC for FPR below 5% is 0.0056 v.s. 0.0064, 0.0022 v.s. 0.0025, 0.0016 v.s. 0.0020, 0.0062 v.s. 0.0062, and 0.0017 v.s. 0.0150 on sponge, coral, hydra, urchin and sea anemone, respectively.

We also evaluated the performance of the two methods using different thresholds. The receiver operating characteristic (ROC) curves in Figure 4 imply that SECOM has an overall improved performance over DIVCLUS. The differences of area under curve (AUC) between SECOM and DIVCLUS were tested using non-parametric bootstrapping by performing 2,000 resampling. The p-values (less than 0.001) suggest significant improvements of SECOM over DIVCLUS. However, at small false positive rates, SECOM has very similar but slightly lower AUC than DIVCLUS, as shown in Figure 4. Note that the unsmooth curves of DIVCLUS on coral and sea anemone are due to the fact that DIVCLUS predicted small numbers of domains on these two proteomes.

### SECOM’s Ability to Predict Putative Novel Domains

SECOM and DIVCLUS predicted, respectively, 1,783 and 1,138 putative novel domains (domains not recovered by InterProScan). Of the 1,138 DIVCLUS predicted domains, 825 are also recovered by SECOM, with average recall and precision values of 84.4% and 96.1%, respectively. The majority of the putative novel domains are thus predicted by both SECOM and DIVCLUS.

We also found 1,015 SECOM domains that were not recovered by either DIVCLUS or InterProScan. To assess whether these domains were putative novel domains or false positives, we used a validation process similar to the one used in [27]. A cluster of segments is likely to be a real domain if the proteins containing these segments have other evidence of similarities; for instance, sharing common domains that are annotated by InterProScan is such evidence. For each of the 1,015 clusters, we annotated all the protein sequences with known domains using InterProScan. We excluded those clusters that contained fewer than two segments to which the corresponding proteins contained annotated domains and those domain clusters in which the predicted domain overlapped with the known domain regions from InterProScan. After this filtering step, 86 clusters remained, 15 of which contained more than four segments. Of these, 78.4% putative novel domains occurred in proteins with at least one known domain. The 10 largest clusters of putative novel domains predicted by SECOM are summarized in Table 3. The most frequent annotated domains in these clusters are usually shared by most of the segments in the clusters, not by segments in different clusters, suggesting that these may be different novel domains.

**Table 3:** Summary of shared annotated domains of the 10 largest clusters detected by SECOM but not covered by InterProScan or DIVCLUS.

Size	#	Domain	%	Description	GO Function
19	19	SSF52540	84.2	P-loop containing nucleoside triphosphate hydrolases	
		PF05729	84.2	NACHT	
		SSF52047	31.6	RNI-like	
		SSF53167	10.5	Purine and uridine phosphorylases	
18	2	IPR011050	11.1	Pectin lyase-like Pectin lyase fold/virulence factor	
11	11	IPR002181	100	Fibrinogen_C Fibrinogen, alpha/beta/gamma chain,C-terminal globular	Molecular Function: receptor binding (GO:0005102), Biological Process: signal transduction (GO:0007165)
11	4	IPR008957	27.3	Fibronectin type III Fibronectin, type III-like fold	
		IPR003961	27.3	fn3 Fibronectin, type III	
9	9	SSF101898	100	NHL repeat	
		PF01436	100	NHL	
		IPR000315	77.8	zf-B_box Zinc finger, B-box	Cellular Component: intracellular (GO:0005622), Molecular Function: zinc ion binding (GO:0008270)
9	9	SSF52540	100	P-loop containing nucleoside triphosphate hydrolases	
		PF05729	100	NACHT	
		SSF52047	22.2	RNI-like	
7	7	SSF52540	71.4	P-loop containing nucleoside triphosphate hydrolases	
		PF05729	71.4	NACHT	
		SSF52047	28.6	RNI-like	
		SSF53167	28.6	Purine and uridine phosphorylases	
6	6	SSF52540	100	P-loop containing nucleoside triphosphate hydrolases	
		IPR020683	83.3	Ankyrin repeat Ankyrin repeat-containing domain	
		PF00023	66.7	Ank Ankyrin repeat	
6	6	IPR020683	100	Ankyrin repeat Ankyrin repeat-containing domain	
		IPR002110	100	Ank Ankyrin repeat	
6	2	PF05970	33.3	DUF889	

The first column lists the size of the clusters. The second column lists the number of protein sequences that have at least one annotated Pfam or Superfamily domain. The third and fourth columns list the most frequent annotated domains and their frequencies in the clusters. The fifth column shows the domain descriptions. The sixth column lists the enriched Gene Ontology (GO) function (if available).

**Figure 5:**
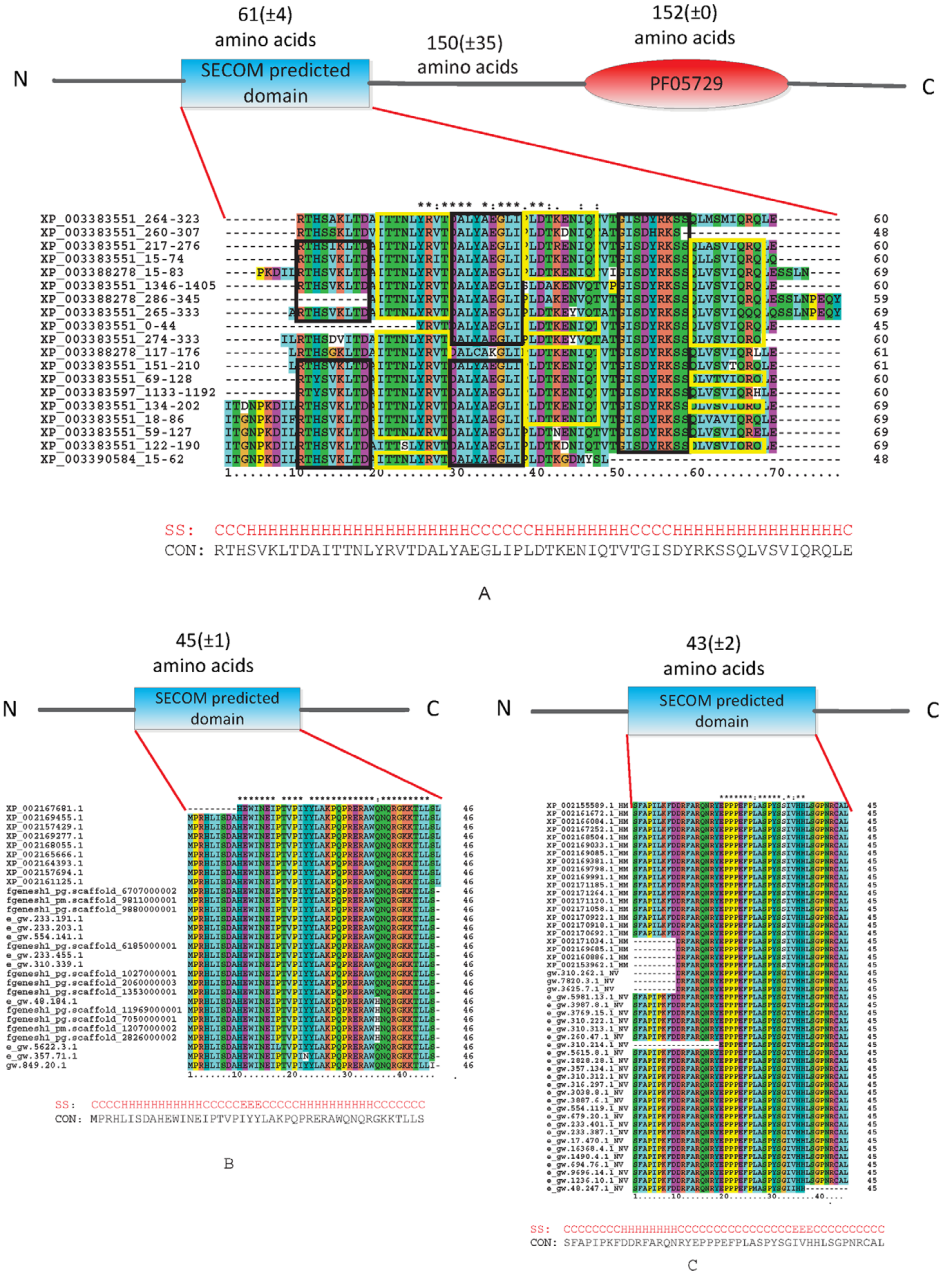
Three putative novel domains predicted by SECOM only. PSIPRED [8] is used to predict the secondary structures of the consensus sequences of the three domains. CON stands for the consensus sequences and SS stands for the predicted secondary structures. (A). ClustalX alignment of the 19 segments. The protein IDs are those of the best BLAST hit in *A. queenslandica* followed by the location of the predicted domain. The hash seeds that correspond to the communities from which the cluster is built are boxed. (B) and (C). ClustalX alignments of two putative novel domains predicted by SECOM only. NV stands for sea anemone and HM stands for hydra.

To further validate the putative novel domains, we selected a domain cluster of size 19 (Tables 3 and S2 and Figure 5 that seemed to contain a novel domain specific to the sponge *Amphimedon queenslandica*. Of the 19 proteins, 16 also have a P-loop containing nucleoside triphosphate hydrolases (PF05729) with 150±35 amino acids after the SECOM predicted domain. We performed BLAST analysis on all the protein sequences in this cluster against the NCBI NR database. For all 19 proteins, the top hits were predicted proteins in *A. queenslandica* and all the proteins matched only four different *A. queenslandica* protein IDs given the database redundancy. The top BLAST hits that were not in *A. queenslandica* were with proteins annotated as “NACHT, LRR and PYD domains-containing protein 10” (13 out of the 16 proteins). NACHT, LRR and PYD domains are usually present in proteins that assembled into the inflammasome once immunological cells recognize the invading pathogens [28]–[30]. The three proteins without the PF05729 domain do not match any NACHT-, LRR- and PYD- containing proteins. We further conducted a multiple sequence alignment of all 19 segments of this predicted domain by using ClustalX [31] (Figure 5(A)). The segments aligned well and the hash seeds identified by SECOM were always aligned to the same columns.

Although several proteins containing a SECOM putative novel domain have at least one known domain, there are still 840 domain clusters remaining unverified. We found 13 putative novel SECOM domains that never appeared in a protein together with any Pfam or Superfamily domains, but that are identified across more than one species. Two examples are discussed here. The first example is present in 20 sea anemone and 9 hydra proteins. The sequence alignment of the 29 segments is shown in Figure 5(B). Interestingly, when searching the Pfam-B database, all 29 proteins hit the domain PB011651, a domain of unknown function found only in bacterial genomes. A perfect hit is found with a protein predicted from marine metagenomic datasets stored in the environmental sample database (http://www.ncbi.nlm.nih.gov/protein/143884714?report=genbank&log=prottop&blast_rank=1&RID=DYMP8HHZ011) (Figure S11), indicating a possible bacterial origin of the sea anemone and hydra proteins by lateral gene transfer [32]. Another interesting example can be found in 28 sea anemone and 21 hydra proteins (Figure 5(C)). Like the previous example, most of the proteins that contain these segments (46 out of 49) are single-domain proteins and no hits are found after searching the Superfamily or Pfam-B databases, but a search of the Pfam-A database retrieves a domain of unknown function also found only in bacteria genomes identified in marine environmental metagenomic projects (http://www.ncbi.nlm.nih.gov/protein/142495124?report=genbank&log=prottop&blast_rank=1&RID=DYMSR9PC016). A BLAST search of the NCBI NR database reveals that this domain is also found in some Cnidarian and Fungal species (Figure S12).

## Discussion

With the increasing availability of new genome sequences for non-model organisms, there is an urgent need for highly efficient and effective tools to annotate and analyze genomes of species for which there is a paucity of sequencing data and functional annotation, particularly if compared to model organisms. Here, we propose SECOM, a powerful, novel tool that automatically identifies protein domains at a genome-wide scale. SECOM is based on the assumption that domains are recurring segments in protein repertoires and are more highly conserved during evolution than are those in the inter-domain regions. Here, we were able to show that these assumptions are reasonable and demonstrate that SECOM is able to infer high coverage of domains predicted by both database- and alignment-based methods.

Furthermore, SECOM shows high sensitivity to detecting putative novel domains, which makes it a valuable tool for comparative genomic studies through which scientists are often searching novel taxonomically restricted proteins defined by species-specific domains or specific combinations of domains. Here, we show that SECOM is able to detect significantly more putative novel domains than DIVCLUS can and discover novel domains in proteins that are already functionally annotated by InterProScan. Using SECOM to analyze five non-model organisms, we are able to find several putative novel domains and to propose the possible origin of these by Lateral Gene Transfer from aquatic microbial communities. Note that SECOM can be used together with any other domain predictors. Given a proteome dataset, the database-based methods, such as InterProScan, can be first applied to detect known domains. The remaining protein subsequences after cutting the InterProScan domains out can then be used as inputs for SECOM, which has been demonstrated to be sensitive to detect novel domains.

Finally, SECOM is several orders of magnitude faster than DIVCLUS. Note that similar to many all-against-all alignment-based domain predictors, DIVCLUS calls the Smith-Waterman algorithm as a subroutine. Thus, the speed of such methods is dominated by the alignment step. Therefore, SECOM is expected to be orders of magnitude faster than the other widely used genome-scale domain predictors and this can be very advantageous in reducing the computational time when analyzing several large genomes in parallel. As shown in Figures S5 and S10, the runtime of SECOM seems to be sublinear to the length of hash seeds and constant to the merging threshold. However, the space complexity increases quickly when longer hash seeds are used. To be more specific, when six is used as the length of hash seeds, 300 Mb of memory is needed, whereas almost 1,000 Mb of memory is needed for seed length nine.

## Supporting Information

Figure S1 **The relationship between the length of the hash seeds and the cluster-level recall and precision of SECOM on the sponge proteome.**
(PDF)

Figure S2 **The relationship between the length of the hash seeds and the in-cluster-level recall and precision of SECOM on the sponge proteome.**
(PDF)

Figure S3 **The relationship between the length of the hash seeds and the revised cluster-level recall and precision of SECOM on the sponge proteome.**
(PDF)

Figure S4 **The relationship between the length of the hash seeds and the revised in-cluster-level recall and precision of SECOM on the sponge proteome.**
(PDF)

Figure S5 **The relationship between the length of the hash seeds, and the runtime and the memory use of SECOM on the sponge proteome.**
(PDF)

Figure S6 **The relationship between the merging threshold and the cluster-level recall and precision of SECOM on the sponge proteome.**
(PDF)

Figure S7 **The relationship between the merging threshold and the in-cluster-level recall and precision of SECOM on the sponge proteome.**
(PDF)

Figure S8 **The relationship between the merging threshold and the revised cluster-level recall and precision of SECOM on the sponge proteome.**
(PDF)

Figure S9 **The relationship between the merging threshold and the revised in-cluster-level recall and precision of SECOM on the sponge proteome.**
(PDF)

Figure S10 **The relationship between the merging threshold and the runtime and the memory use of SECOM on the sponge proteome.**
(PDF)

Figure S11 **The BLAST taxonomy report for the putative novel domain that contains 29 segments.**
(PNG)

Figure S12 **The BLAST taxonomy report for the putative novel domain that contains 49 segments.**
(PNG)

Table S1 **Overall performance of SECOM and DIVCLUS on the five aquatic proteomes.** The revised recall, precision and F1 score values are given in percentiles. DIV denotes DIVCLUS and SEC denotes SECOM.
(TEX)

Table S2 **An example of a putative novel domain cluster.** Of these 19 segments, 16 have a P-loop containing nucleotide triphosphate hydrolases domain (PF05729). The first column lists protein ID of the best hit for *A. queenslandica*. The second column lists the positions of the segments identified by SECOM on the protein sequences. The third column lists the distance of the SECOM predicted domains to the PF05729 domains. In the fourth column is the description of the top five BLAST hits that are not *A. queenslandica* proteins.
(TEX)

Materials S1 **Supplemental methods.**
(PDF)

## References

[1] Pennacchio LA, Rubin EM (2001). Genomic strategies to identify mammalian regulatory sequences.. Nature Reviews Genetics.

[2] Rose GD (1979). Hierarchic organization of domains in globular proteins.. Journal of Molecular Biology.

[3] Wetlaufer DB (1973). Nucleation, rapid folding, and globular intrachain regions in proteins.. Proceedings of the National Academy of Sciences of the United States of America.

[4] King N, Westbrook MJ, Young SL, Kuo A, Abedin M (2008). The genome of the choanoagellate monosiga brevicollis and the origin of metazoans.. Nature.

[5] Srivastava M, Begovic E, Chapman J, Putnam NH, Hellsten U (2008). The trichoplax genome and the nature of placozoans.. Nature.

[6] Baker D, Sali A (2001). Protein structure prediction and structural genomics.. Science.

[7] Berman HM, Bhat TN, Bourne PE, Feng Z, Gilliland G (2000). The protein data bank and the challenge of structural genomics.. Nature Structural & Molecular Biology.

[8] Jones DT (1999). Protein secondary structure prediction based on position-specific scoring matrices.. Journal of Molecular Biology.

[9] Ward JJ, McGuffin LJ, Buxton BF, Jones DT (2003). Secondary structure prediction with support vector machines.. Bioinformatics.

[10] Cheng J, Baldi P (2007). Improved residue contact prediction using support vector machines and a large feature set.. BMC Bioinformatics.

[11] Karplus K, Barrett C, Hughey R (1998). Hidden markov models for detecting remote protein homologies.. Bioinformatics.

[12] Tress M, Cheng J, Baldi P, Joo K, Lee J (2007). Assessment of predictions submitted for the CASP7 domain prediction category.. PROTEINS.

[13] Park J, Teichmann S (1998). Divclus: an automatic method in the geanfammer package that finds homologous domains in single-and multi-domain proteins.. Bioinformatics.

[14] Sonnhammer EL, Kahn D (1994). Modular arrangement of proteins as inferred from analysis of homology.. Protein Science.

[15] Li W, Ma B, Zhang K (2009). Amino acid classi_cation and hash seeds for homology search.. Bioinformatics and Computational Biology.

[16] Newman MEJ, Girvan M (2004). Finding and evaluating community structure in networks.. Physical Review E.

[17] Palla G, Derenyi I, Farkas I, Vicsek T (2005). Uncovering the overlapping community structure ofcomplex networks in nature and society.. Nature.

[18] Kumpula JM, Kivela M, Kaski K, Saramaki J (2008). Sequential algorithm for fast clique percolation.. Physical Review E.

[19] Srivastava M, Simakov O, Chapman J, Fahey B, Gauthier M (2010). The amphimedon queenslandica genome and the evolution of animal complexity.. Nature.

[20] Chapman J, Kirkness E, Simakov O, Hampson S, Mitros T (2010). The dynamic genome of hydra.. Nature.

[21] Putnam N, Srivastava M, Hellsten U, Dirks B, Chapman J (2007). Sea anemone genome reveals ancestral eumetazoan gene repertoire and genomic organization.. Science.

[22] Sodergren E, Weinstock G, Davidson E, Cameron R, Gibbs R (2006). The genome of the sea urchin strongylocentrotus purpuratus.. Science.

[23] Meyer E, Aglyamova G, Wang S, Buchanan-Carter J, Abrego D (2009). Sequencing and de novo analysis of a coral larval transcriptome using 454 gsx.. BMC Genomics.

[24] Apweiler R, Attwood T, Bairoch A, Bateman A, Birney E (2001). The interpro database, an integrated documentation resource for protein families, domains and functional sites.. Nucleic Acids Research.

[25] Ma B, Tromp J, Li M (2002). Patternhunter: faster and more sensitive homology search.. Bioinformatics.

[26] Li M, Ma B, Kisman D, Tromp J (2004). Patternhunter ii: highly sensitive and fast homology search.. Journal of Bioinformatics and Computational Biology.

[27] Enright A, Van Dongen S, Ouzounis C (2002). An efficient algorithm for large-scale detection of protein families.. Nucleic Acids Research.

[28] Tschopp J, Martinon F, Burns K (2003). Nalps: a novel protein family involved in inammation.. Nature Reviews Molecular Cell Biology.

[29] Inohara N, Nuñez G (2003). Nods: intracellular proteins involved in inammation and apoptosis.. Nature Reviews Immunology.

[30] Wang Y, Hasegawa M, Imamura R, Kinoshita T, Kondo C (2004). Pynod, a novel apaf-1/ced4-like protein is an inhibitor of asc and caspase-1.. International immunology.

[31] Chenna R, Sugawara H, Koike T, Lopez R, Gibson T (2003). Multiple sequence alignment with the clustal series of programs.. Nucleic Acids Research.

[32] Blanchard J, Lynch M (2000). Organellar genes: why do they end up in the nucleus?. Trends in Genetics.

