# Supplementary figures and images for: SECOM: A Novel Hash Seed and Community Detection Based-Approach for Genome-Scale Protein Domain Identification
Source: PLoS One. 2012 Jun 28;7(6):e39475. doi: 10.1371/journal.pone.0039475 (PMC3386278; doi:10.1371/journal.pone.0039475)

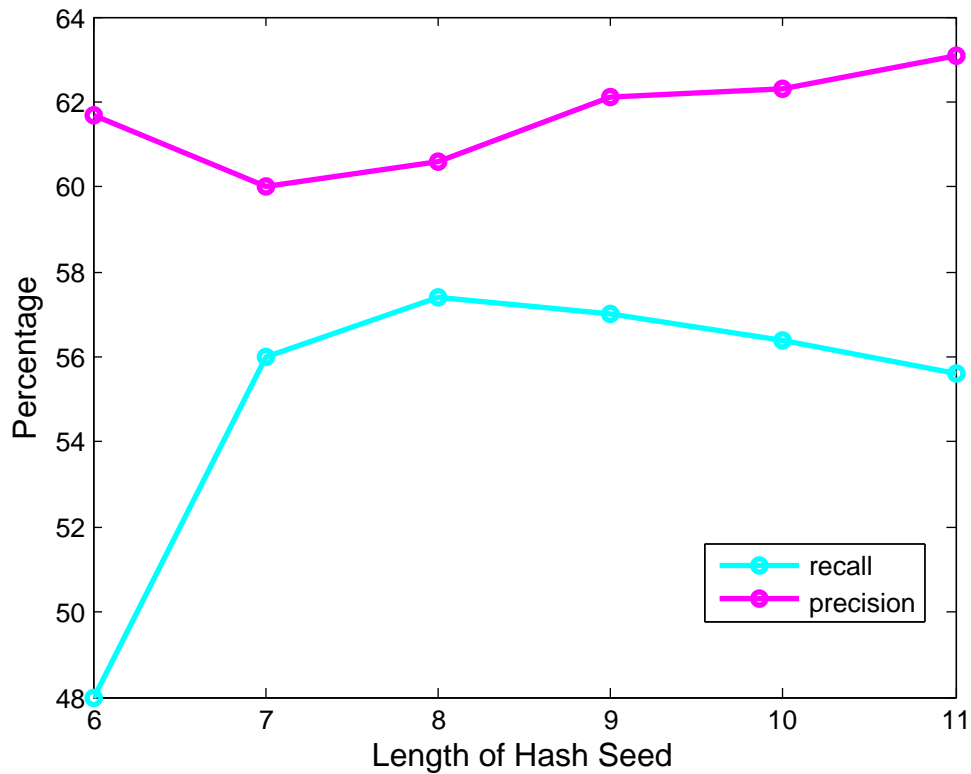

Supplement: Figure S1 — The relationship between the length of the hash seeds and the cluster-level recall and precision of SECOM on the sponge proteome. (PDF) [file pone.0039475.s001.pdf]

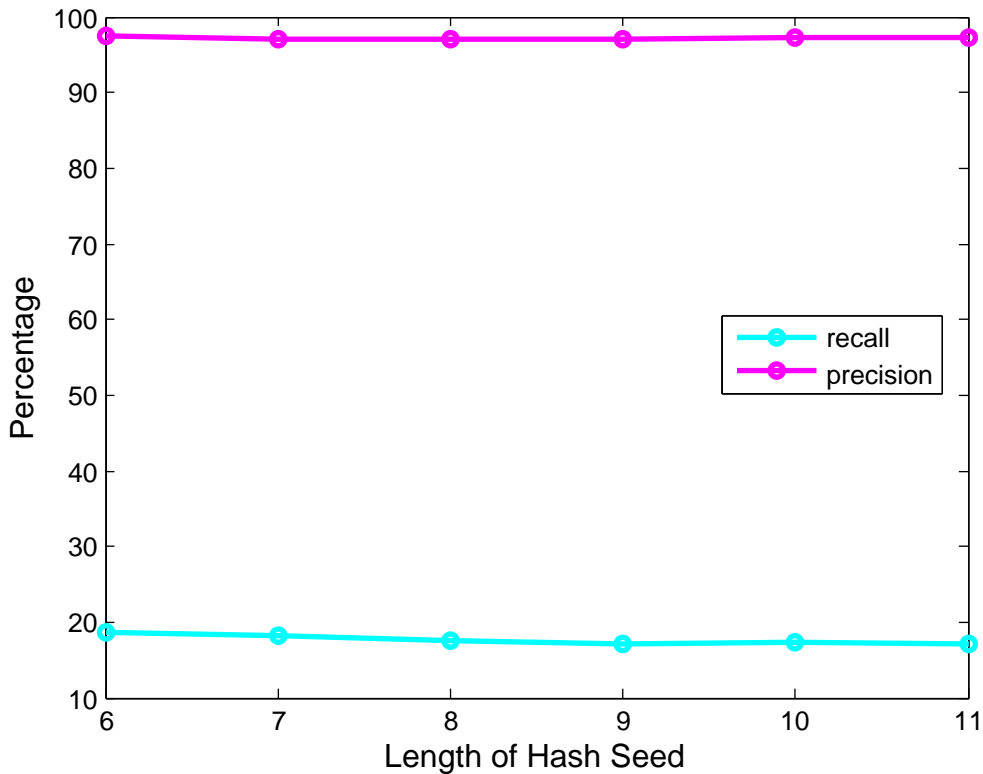

Supplement: Figure S2 — The relationship between the length of the hash seeds and the in-cluster-level recall and precision of SECOM on the sponge proteome. (PDF) [file pone.0039475.s002.pdf]

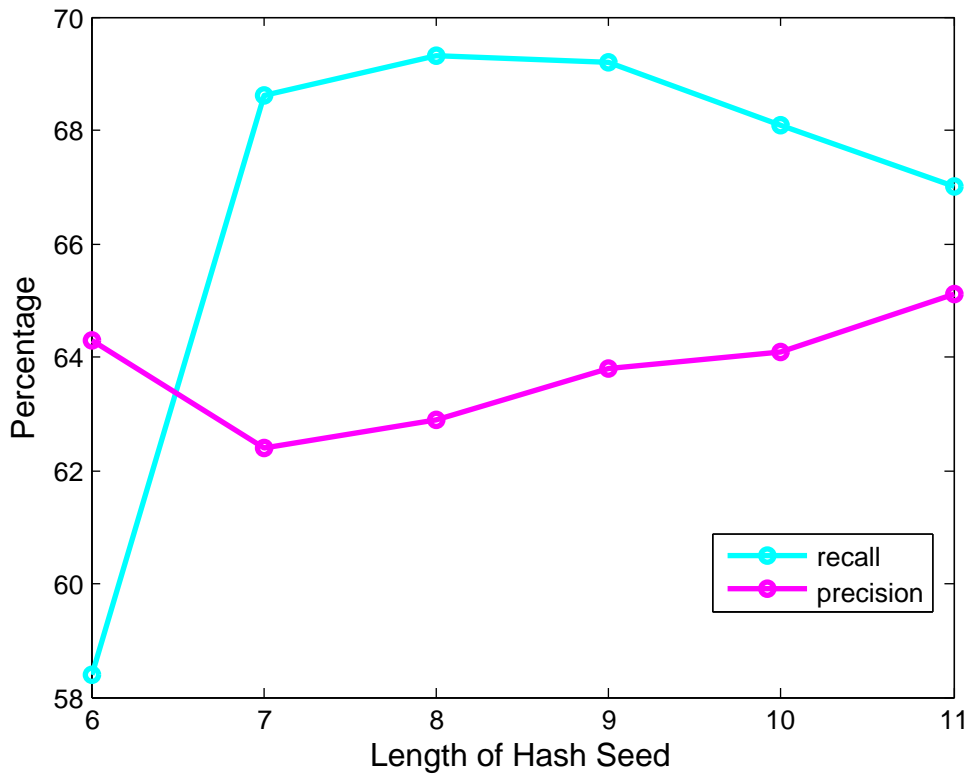

Supplement: Figure S3 — The relationship between the length of the hash seeds and the revised cluster-level recall and precision of SECOM on the sponge proteome. (PDF) [file pone.0039475.s003.pdf]

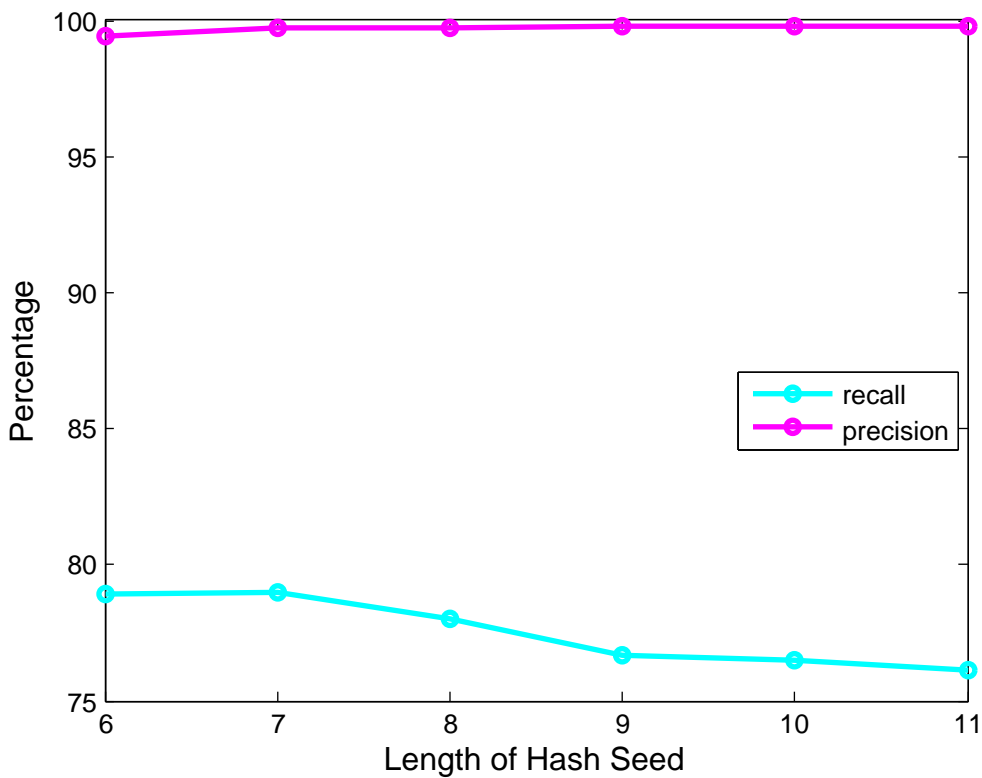

Supplement: Figure S4 — The relationship between the length of the hash seeds and the revised in-cluster-level recall and precision of SECOM on the sponge proteome. (PDF) [file pone.0039475.s004.pdf]

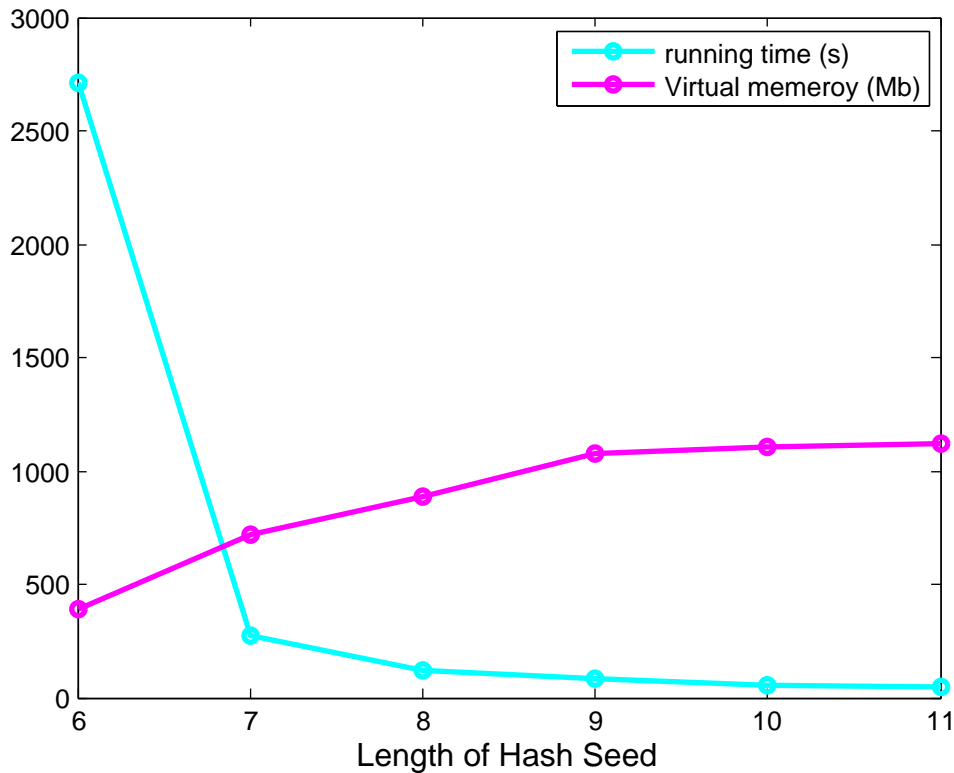

Supplement: Figure S5 — The relationship between the length of the hash seeds, and the runtime and the memory use of SECOM on the sponge proteome. (PDF) [file pone.0039475.s005.pdf]

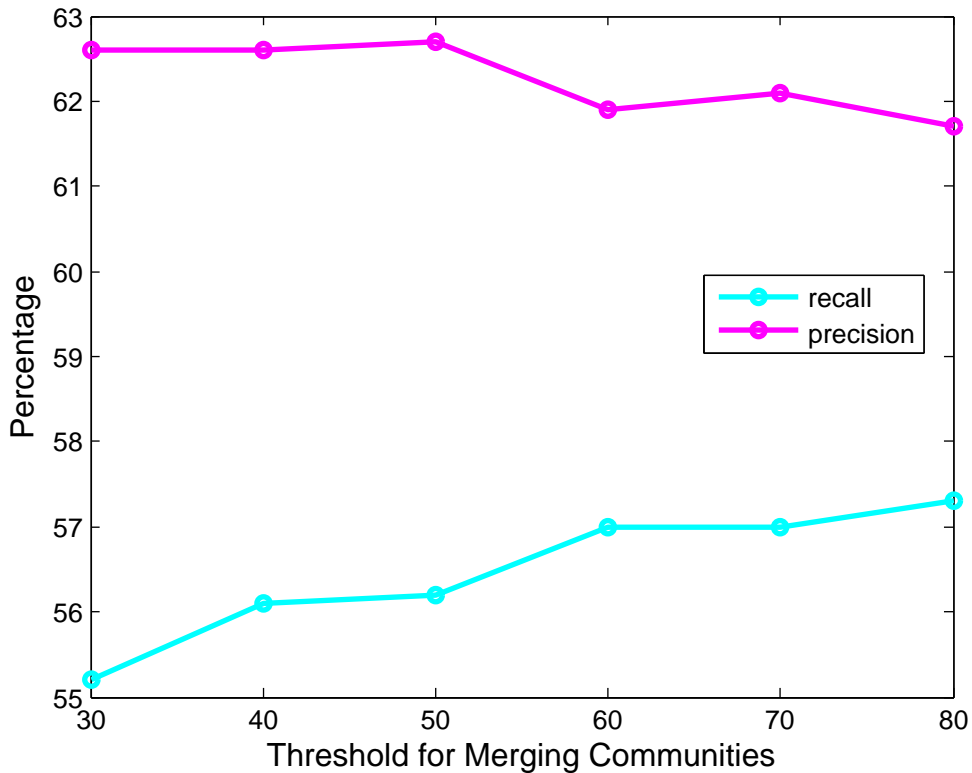

Supplement: Figure S6 — The relationship between the merging threshold and the cluster-level recall and precision of SECOM on the sponge proteome. (PDF) [file pone.0039475.s006.pdf]

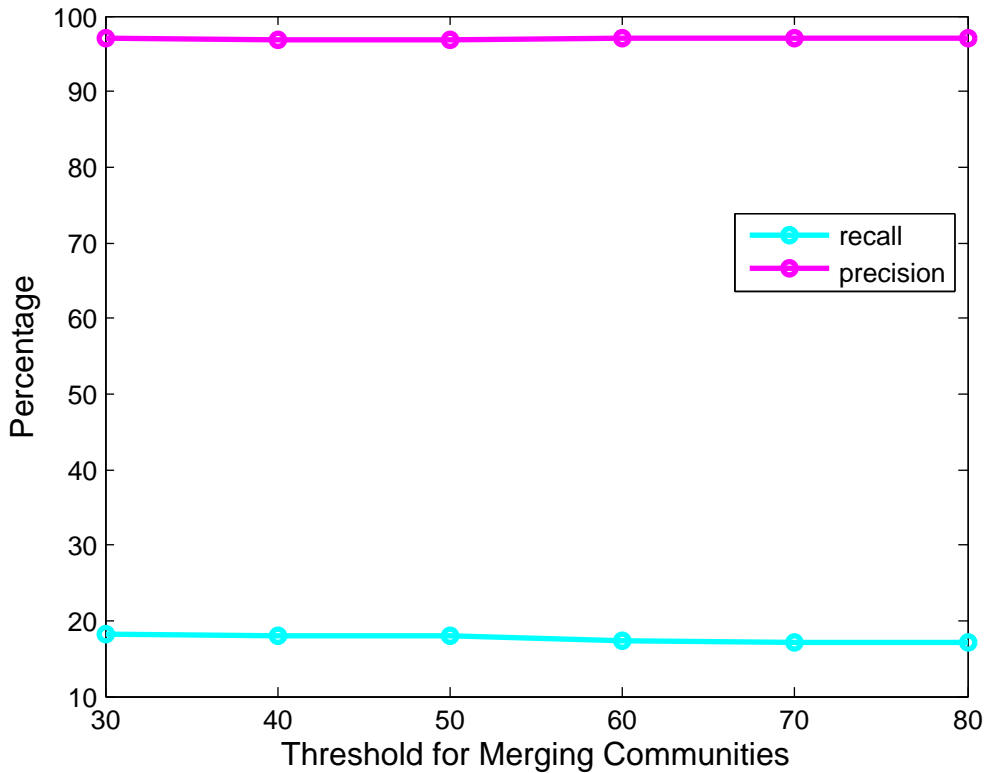

Supplement: Figure S7 — The relationship between the merging threshold and the in-cluster-level recall and precision of SECOM on the sponge proteome. (PDF) [file pone.0039475.s007.pdf]

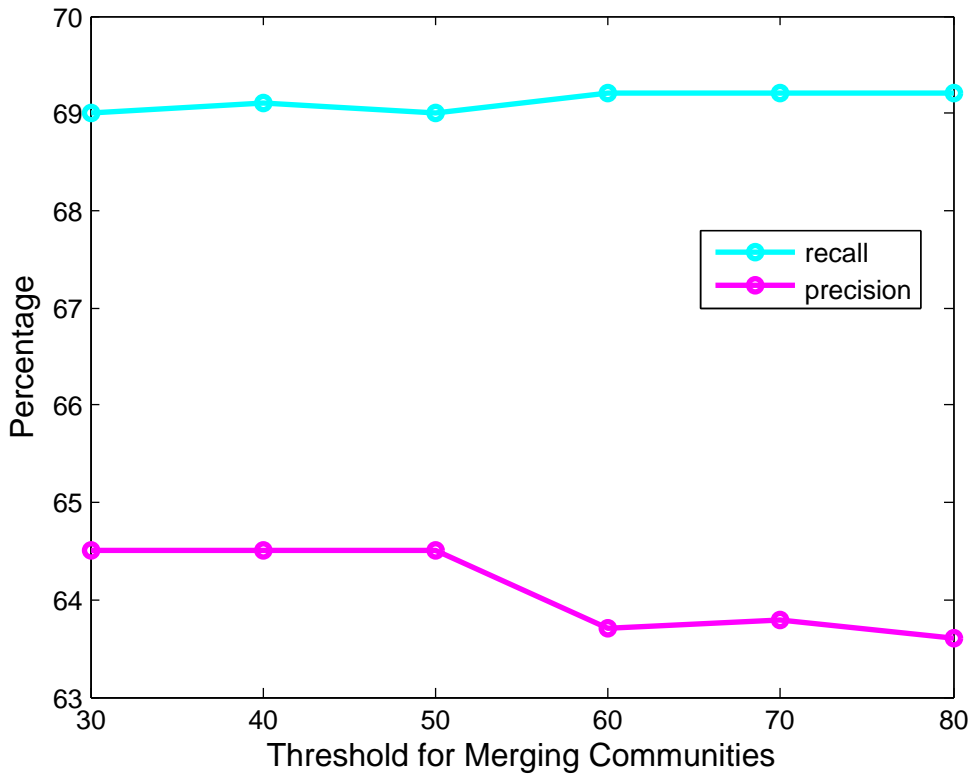

Supplement: Figure S8 — The relationship between the merging threshold and the revised cluster-level recall and precision of SECOM on the sponge proteome. (PDF) [file pone.0039475.s008.pdf]

Percentage

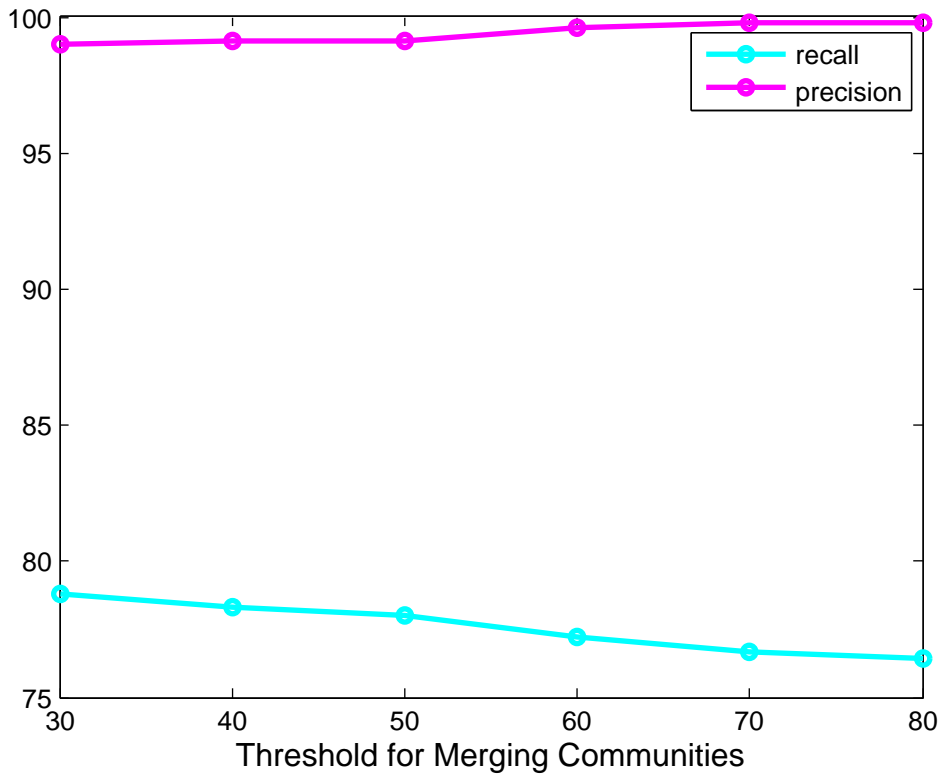

Supplement: Figure S9 — The relationship between the merging threshold and the revised in-cluster-level recall and precision of SECOM on the sponge proteome. (PDF) [file pone.0039475.s009.pdf]

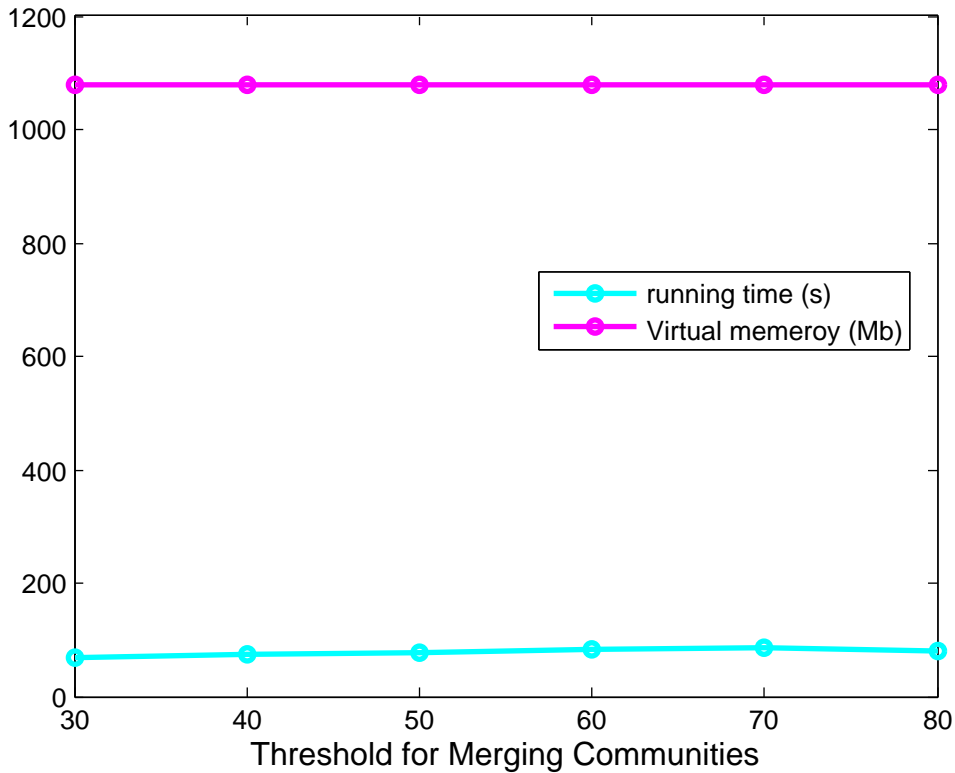

Supplement: Figure S10 — The relationship between the merging threshold and the runtime and the memory use of SECOM on the sponge proteome. (PDF) [file pone.0039475.s010.pdf]

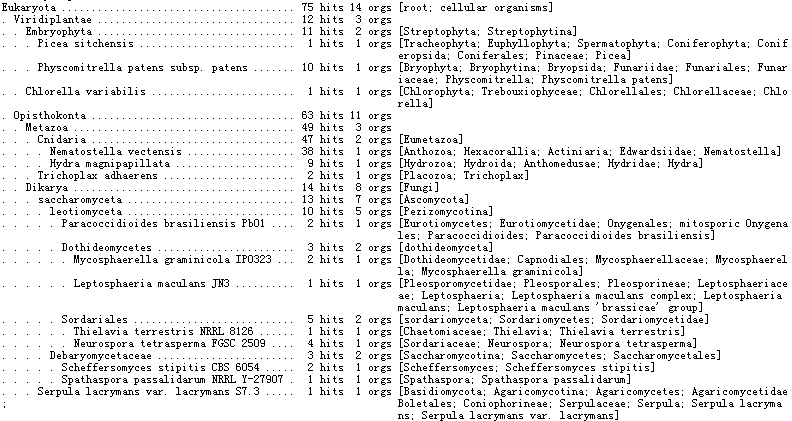

Supplement: Figure S11 — The BLAST taxonomy report for the putative novel domain that contains 29 segments. (PNG) [file pone.0039475.s011.png]

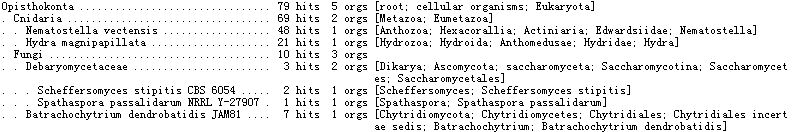

Supplement: Figure S12 — The BLAST taxonomy report for the putative novel domain that contains 49 segments. (PNG) [file pone.0039475.s012.png]
