# Supplementary material for: SECOM: A Novel Hash Seed and Community Detection Based-Approach for Genome-Scale Protein Domain Identification
Source: PLoS One. 2012 Jun 28;7(6):e39475. doi: 10.1371/journal.pone.0039475 (PMC3386278; doi:10.1371/journal.pone.0039475)
Supplement: Materials S1 — Supplemental methods. (PDF) [file pone.0039475.s015.pdf]

Supplementary Materials

Methods

Hash Seeds and Hash Function

Let C be an amino acid classification by rearranging the amino acids in the BLOSUM62 matrix such that the positive values are concentrated around the diagonals and assigning an integer to each class [1].

Let Σ be the set of alphabets of all amino acids. A classification is defined as $C = \{c_1, c_2, \cdots, c_w\}$. Each c_i is a subset of Σ that satisfies $c_i \cap c_j = \emptyset$ for $i \neq j$, and $\cup_{i=1}^{w} c_i = \Sigma$ where $w = |C|$ denotes the number of different classes of the classification. There are two classifications proposed in [1], namely C^1 and C^2 with $w = 11$ and 15, respectively. SECOM uses C^2 as the default setting because C^2 is more sensitive than C^1 according to [1]. More specifically, we use $c(a)$ to denote the class to which an amino acid a belongs.

Let $s = (a_1, a_2, \cdots, a_n)$ be a fragment of length n, which we refer to as an n-mer. The n-mer under classification C is represented as $c(a_1)c(a_2)\cdots c(a_n)$. That is, an n-mer can be represented by a string of amino acid classes, which are assigned by different integers. Each input protein sequence is divided into n-mers by a sliding window of size n and step size one. Each n-mer is called a hash seed. Therefore, a protein sequence with l amino acids should have $l - n + 1$ hash seeds.

We use Rabin-Karp's method [2] to compute the hash values of the successive hash seeds of a protein sequence efficiently. One popular and effective rolling hash function treats every substring as a number in base b, where the base is usually chosen as a large prime number. The hash function is defined as

$$h(s) = c(a_1) \times b^{n-1} + c(a_2) \times b^{n-2} + \cdots c(a_n) \times b^0 \quad (1)$$

Suppressing the Sliding Window Effects of Hash Seeds

The sliding window effects appear when a highly conserved segment recurs in more than one protein. Let $S = \{s_1, s_2, \cdots, s_t\}$ be a set of hash seeds generated from a protein sequence, where s_i represents the starting position of the $i-$th hash seed in this protein. A hit to S is detected if there is a hash seed with the same hash value in either the same protein or another protein. Let $H_S = \{h_{i_1}, h_{i_2}, \cdots, h_{i_q}\}$ ($\{h_{i_1}, h_{i_2}, \cdots, h_{i_q}\} \subset \{s_1, s_2, \cdots, s_t\}$) be all the hits for S. From our definition, a hash seed represents a highly conserved fragment of length n. Therefore, an overlap exists between two hits h_i and h_j when $abs(h_i - h_j) < n$. In this case, a highly conserved segment can generate a set of sliding window hits, which will result in a huge number of uninformative seeds. To avoid this effect, we assume that two successive hits in one protein sequence must be at least n amino acids apart. That is, $abs(h_i - h_j) \geq n$ for any pair of hits on a certain protein sequence.

Confidence Score of Domain Clusters

SECOM assigns a confidence score for every predicted domain cluster. The confidence score is defined as the normalized domain size times the average hash seed density as

$$Score_{SEC} = \frac{|D|}{|D_{max}|} \times \sum_{d_i \in D} \frac{n \times p_i}{|d_i|}, \quad (2)$$

where D denotes the domain cluster, $|D|$ is the number of segments in D, $|D_{max}|$ is the number of segments in the largest domain cluster, d_i denotes any segment in the cluster, $|d_i|$ is the length of the segment, n denotes the length of the hash seed, and p_i denotes the number of hash seeds in d_i. Apparently, in the ideal case, the confidence score is 1. Given a threshold between 0 and 1, if the confidence score is higher than the threshold, the domain cluster is considered to be a prediction.

Evaluation Criteria

Both SECOM and DIVCLUS are applied to the five encoded proteomes. The predicted domains are compared with those annotated in InterProScan, which to our knowledge, is the most complete and comprehensive domain database. We evaluate the performance of both methods vs. InterProScan by using four criteria, i.e., recall, precision, F1 score, and runtime.

- *Recall and precision*
  A cluster of protein segments represents each predicted domain in InterProScan, SECOM or DIVCLUS. We measure two kinds of recall and precision in order to evaluate the performance of a method. First, we need to know how many protein clusters, i.e., domains, annotated by InterProScan are also predicted by SECOM or DIVCLUS. Second, we need to know how many segments in an InterProScan cluster are members of the matched cluster predicted by SECOM or DIVCLUS.

  The first kind of recall and precision is referred to as $recall_{clu}$ and $precision_{clu}$, and the second kind is referred to as $recall_{inClu}$ and $precision_{inClu}$. For both kinds of notation, recall and precision are defined as

$$recall = \frac{TP}{TP + FN},$$
$$precision = \frac{TP}{TP + FP},$$

  where TP signifies true positive, which is the number of predictions that are true, FN signifies false negative, which is the number of true predictions that are not predicted, and FP signifies false positive, which is the number of predictions that are false. In our evaluation, two clusters are considered to be a match if they share at least 50% of the matched segments. Two segments are considered to be a match if they cover the same protein sequence.

- *F1 score*
  There is always a tradeoff between recall and precision. Therefore, we further calculate the F1 score to assess the method's balance. The F1 score is calculated as the harmonic mean of recall and precision

$$F1 = \frac{2 \cdot recall \cdot precision}{recall + precision} \tag{3}$$

- *Runtime*
  SECOM is a hash seed based domain detection method that does not need to conduct pairwise alignment. It has a nearly-linear runtime to the size of the inputs, rather than the quadratic runtime of DIVCLUS. Therefore, SECOM is supposed should be much faster than DIVCLUS.

Validation Datasets

We conducted the validation procedure on five recently sequenced aquatic animals. The sponge genome sequence was reported in [3], which was sequenced from *A. queenslandica*, a demosponge from the Great Barrier Reef. The hydra genome sequence was reported in [4], which was sequenced from *Hydra magnipapillata*. The sea anemone genome sequence was reported in [5], which was sequenced from the starlet sea anemone *Nematostella vectensis*. The sea urchin genome sequence was reported in [6], which was sequenced from the sea urchin *Strongylocentrotus purpuratus*. The numbers of protein sequences extracted from the sponge, hydra, anemone and urchin were 30,327, 17,398, 27,273 and 42,420, respectively. Finally, the protein sequences for coral (69,160 proteins) were not from genome sequencing. Instead, they were from the transcriptome of the reef-building coral *Acropora millepora* [7], and coding sequences were identified as described in [8].

Results

Effects of Seed Length

SECOM uses nine as the default length of the hash seeds. Intuitively, if the length of the hash seeds is short, more hits will be discovered. However, the short seeds are less reliable than the long seeds, which means that the communities formed by the short seeds may contain more false positives. When evaluating the cluster-level recall and precision, we require a certain percentage of hits between two clusters. The large number of false positives introduced by the short seeds may reduce the number of matched clusters, and thus cause lower recall and precision values. On the other hand, if the seed is too long, a significantly smaller number of hits will be discovered, which will also cause low recall and precision values. Therefore, there should be a tradeoff length for the hash seeds.

As shown in Figures S1 and S3, the cluster-level recall achieves peak values for seed length of eight and nine. The in-cluster-level recall and precision, on the other hand, do not change much for different seed lengths, as shown in Figures S2 and S4. When the length of the hash seed increases, the running time decreases but the memory increases (Figure S5).In SECOM, we select nine as the default value for the hash seeds. The users have the option to choose different seed lengths as the parameter for the program.

Effects of Merging Threshold

SECOM uses 70% as the threshold for merging two communities. We further test the effect of this threshold by evaluating the performance of SECOM using different merging thresholds. As shown in Figures S6 and S8, when the merging threshold increases, the cluster-level recall value increases but the precision value decreases. For the in-cluster performance, when the merging threshold increases, the recall and precision do not change by much (Figures S7 and S9).Furthermore, the merging threshold does not have an obvious effect on the runtime and the memory of SECOM, as shown in Figure S10.

Revised Performance of SECOM and DIVCLUS on Aquatic Proteomes

The performance of SECOM and DIVCLUS on all five aquatic proteomes is shown in Table S1. SECOM outperforms DIVCLUS on all criteria, except for the cluster-level precision. It can be seen that 64% of the domains annotated by InterProScan are also detected by SECOM, whereas inside each domain cluster, more than 88% of the segments from InterProScan are covered by SECOM as well. This clearly demonstrates the ability of SECOM to detect the known domains. The domains or segments which are annotated by InterProScan but not detected by SECOM either do not have high sequential similarity or require structural information to detect them.

An Example of a Putative Novel Domain

The details of an example of a putative novel domain that contains 19 segments are listed in Table S2.

Taxonomy Report of the Two Novel Domains

The NCBI taxonomy reports for the two putative novel domains (one with 29 segments and one with 49 segments) are shown in Figures S11 and S12.

References

[1] Li W, Ma B, Zhang K (2009) Amino acid classification and hash seeds for homology search. Bioinformatics and Computational Biology 5462: 44-51.

[2] Karp RM, Rabin MO (1987) Efficient randomized pattern-matching algorithms. IBM Journal of Research and Development 31: 249-260.

[3] Srivastava M, Simakov O, Chapman J, Fahey B, Gauthier M, et al. (2010) The amphimedon queenslandica genome and the evolution of animal complexity. Nature 466: 720–726.

[4] Chapman J, Kirkness E, Simakov O, Hampson S, Mitros T, et al. (2010) The dynamic genome of hydra. Nature 464: 592–596.

[5] Putnam N, Srivastava M, Hellsten U, Dirks B, Chapman J, et al. (2007) Sea anemone genome reveals ancestral eumetazoan gene repertoire and genomic organization. Science 317: 86-94.

[6] Sodergren E, Weinstock G, Davidson E, Cameron R, Gibbs R, et al. (2006) The genome of the sea urchin strongylocentrotus purpuratus. Science 314: 941-952.

[7] Meyer E, Aglyamova G, Wang S, Buchanan-Carter J, Abrego D, et al. (2009) Sequencing and de novo analysis of a coral larval transcriptome using 454 gsflx. BMC Genomics 10: 219.

[8] Ryu T, Mavromatis C, Bayer T, Voolstra C, Ravasi T (2011) Unexpected complexity of the reef-building coral acropora millepora transcription factor network. BMC Systems Biology 5: 58.
